# Transient CD4^+^ T Cell Depletion Results in Delayed Development of Functional Vaccine-Elicited Antibody Responses

**DOI:** 10.1128/JVI.00039-16

**Published:** 2016-04-14

**Authors:** Nicholas M. Provine, Alexander Badamchi-Zadeh, Christine A. Bricault, Pablo Penaloza-MacMaster, Rafael A. Larocca, Erica N. Borducchi, Michael S. Seaman, Dan H. Barouch

**Affiliations:** a Center for Virology and Vaccine Research, Beth Israel Deaconess Medical Center, Harvard Medical School, Boston, Massachusetts, USA; b Ragon Institute of MGH, MIT, and Harvard, Boston, Massachusetts, USA

## Abstract

We have recently demonstrated that CD4^+^ T cell help is required at the time of adenovirus (Ad) vector immunization for the development of functional CD8^+^ T cell responses, but the temporal requirement for CD4^+^ T cell help for the induction of antibody responses remains unclear. Here we demonstrate that induction of antibody responses in C57BL/6 mice can occur at a time displaced from the time of Ad vector immunization by depletion of CD4^+^ T cells. Transient depletion of CD4^+^ T cells at the time of immunization delays the development of antigen-specific antibody responses but does not permanently impair their development or induce tolerance against the transgene. Upon CD4^+^ T cell recovery, transgene-specific serum IgG antibody titers develop and reach a concentration equivalent to that in undepleted control animals. These delayed antibody responses exhibit no functional defects with regard to isotype, functional avidity, expansion after boosting immunization, or the capacity to neutralize a simian immunodeficiency virus (SIV) Env-expressing pseudovirus. The development of this delayed transgene-specific antibody response is temporally linked to the expansion of *de novo* antigen-specific CD4^+^ T cell responses, which develop after transient depletion of CD4^+^ T cells. These data demonstrate that functional vaccine-elicited antibody responses can be induced even if CD4^+^ T cell help is provided at a time markedly separated from the time of vaccination.

**IMPORTANCE** CD4^+^ T cells have a critical role in providing positive help signals to B cells, which promote robust antibody responses. The paradigm is that helper signals must be provided immediately upon antigen exposure, and their absence results in tolerance against the antigen. Here we demonstrate that, in contrast to the current model that the absence of CD4^+^ T cell help at priming results in long-term antibody nonresponsiveness, antibody responses can be induced by adenovirus vector immunization or alum-adjuvanted protein immunization even if CD4^+^ T cell help is not provided until >1 month after immunization. These data demonstrate that the time when CD4^+^ T cell help signals must be provided is more dynamic and flexible than previously appreciated. These data suggest that augmentation of CD4^+^ T cell helper function even after the time of vaccination can enhance vaccine-elicited antibody responses and thereby potentially enhance the immunogenicity of vaccines in immunocompromised individuals.

## INTRODUCTION

CD4^+^ T cells, also termed T helper cells, are critical positive regulators of antibody and cytotoxic CD8^+^ T cell responses (1). In the context of antibody induction, the primary function of CD4^+^ T cells is to promote and maintain B cell germinal center responses (2, 3). The current model is that CD4^+^ T cell help must be provided at the time of antigen exposure (by either infection, immunization, or exposure to self-antigen), as an absence of CD4^+^ T cell help at the time of priming results in tolerance (3–9). For productive antibody responses to develop, engagement of the CD40 signaling pathway on B cells by CD4^+^ T cells must occur (5, 10, 11). Studies using model antigens have demonstrated that the proper development of germinal center responses is a dynamic process where CD4^+^ T cell help, via CD40, is provided for several days (8, 12–14). In the absence of these positive signals from CD4^+^ T cells, tolerance is induced because activated B cells are rapidly deleted (2, 10, 15). However, in some, but not all, cases, an antibody response can be induced by readministration of the antigen after recovery of the CD4^+^ T cell population (5, 8, 11, 13). Thus, our current understanding is that CD4^+^ T cell help is required immediately at the time of antigen exposure for the development of functional antibody responses.

Adenovirus (Ad) vectors have primarily been pursued as vaccine platforms due to their ability to induce strong CD8^+^ T cell responses and antibody responses (16–22). We have recently described that following Ad vector immunization of mice, CD4^+^ T cell help is required immediately upon antigen exposure to prevent immediate and irreversible dysfunction of vaccine-elicited CD8^+^ T cells (N. M. Provine, R. A. Larocca, M. Aid, P. Penaloza-MacMaster, A. Badamchi-Zadeh, E. N. Borducchi, K. B. Yates, P. Abbink, M. Kirilova, D. Ng'ang'a, J. Bramson, and D. H. Barouch, submitted for publication). Additionally, this CD4^+^ T cell help is required for a month postimmunization to properly induce CD8^+^ T cell responses (23). However, a role for CD4^+^ T cells in regulating transgene-specific antibody responses following Ad vector immunization has not been previously demonstrated. Thus, we sought to identify a role for CD4^+^ T cells in the promotion of transgene-specific antibody responses following Ad vector immunization of C57BL/6 mice. Furthermore, we sought to determine whether Ad vector vaccine-elicited antibody responses are also immediately and irreversibly dysfunctional if CD4^+^ T cell help is not provided at the time of immunization.

In this study, we identified that following Ad vector immunization, CD4^+^ T cell help is required for between 10 and 14 days after immunization to induce optimal antigen-specific antibody titers. Unexpectedly, we also observed that CD4^+^ T cell depletion prior to immunization does not result in a permanent ablation of antigen-specific antibody responses. Instead, the induction of antibody responses is simply delayed until the time at which the CD4^+^ T cells begin to recover, and these responses developed without the readministration of antigen. These delayed antibody responses exhibit no apparent functional defects, and the development of these responses coincides with the development of delayed antigen-specific CD4^+^ T cell responses. Delayed antigen-specific antibody responses are also observed by using a protein immunogen formulated in an alum-based adjuvant. Thus, in contrast to the current model of an immediate requirement for CD4^+^ T cell help, we demonstrate that functional antibody responses can be induced at a time separate from the time of immunization with Ad vector- or protein-based vaccines in the context of transient CD4^+^ T cell depletion.

## MATERIALS AND METHODS

### Mice, vectors, proteins, and viruses.

Six- to ten-week-old C57BL/6, B6.129S2-H2^dlAb1-Ea^/J (major histocompatibility complex class II [MHC-II] knockout [KO]), B6.129S2-Cd40lg^tm1Imx^/J (CD40L KO), and B6.129P2-Cd40^tm1Kik^/J (CD40 KO) mice were purchased from the Jackson Laboratory (Bar Harbor, ME). Thymectomized C57BL/6 mice underwent adult thymectomy at the Jackson Laboratory (Bar Harbor, ME). Mice were immunized intramuscularly (i.m.) with a volume of 100 μl divided between the two quadriceps. The previously described E1/E3-deleted Ad26-SIV_mac239_ Env or Ad5-SIV_mac239_ Env was used at a dose of 10^9^ or 10^10^ viral particles (vp) (24). Simian immunodeficiency virus (SIV) Env 32H gp140 was used at 50 μg with 100 μg Adju-Phos (Brenntag) (25). All animal experiments were performed in accordance with Institutional Animal Care and Use Committee guidelines of the Beth Israel Deaconess Medical Center.

### Monoclonal antibodies.

The monoclonal anti-CD4 antibody (GK1.5; BioXcell) was administered by two intraperitoneal injections of 500 μg on consecutive days. To maintain depletion of CD4^+^ T cells, where applicable, GK1.5 was readministered in two 500-μg doses every 14 to 21 days.

### Serum collection and tissue processing.

Blood was collected, and serum was clarified by collection of the supernatant following centrifugation at 10,000 rpm for 5 min. Two centrifugation steps were performed, and following clarification, serum was stored at −20°C for further analysis. Splenic and iliac lymph node (LN) mononuclear cells were harvested as previously described (26). Tissues were ground through a 70-μm strainer (Fisher Scientific), red blood cells were lysed by ammonium-chloride-potassium (ACK) treatment for 3 min, and debris was removed by subsequent filtering through a 30-μm filter (Miltenyi Biotec).

### Endpoint ELISA.

Endpoint enzyme-link immunosorbent assays (ELISAs) were performed as previously described (27). Briefly, ELISA plates were coated overnight with 1 μg/ml of SIV_mac239_ gp140 Env. Plates were blocked for 4 h with a solution containing phosphate-buffered saline (PBS), 2% bovine serum albumin (BSA), and 0.05% Tween 20. Mouse serum was serially diluted, added to the ELISA plate, and incubated for 1 h. Bound serum was detected by 1 h of incubation with peroxidase-conjugated, affinity-purified rabbit anti-mouse secondary antibody diluted 1:2,000 (Jackson ImmunoResearch Laboratories). Plates were developed and read on a SpectraMax Plus ELISA plate reader using SoftMax Pro 4.7.1 software (Molecular Devices). Positive titers were defined as the greatest serum dilution with an optical density (OD) >2-fold above the naive negative-control serum OD.

### Isotype and urea disruption ELISAs.

Isotype and urea disruption ELISAs were performed as previously described (28, 29). A semiquantitative immunoglobulin ELISA protocol described previously was followed. Briefly, ELISA plates coated with 0.5 μg/ml SIV_mac239_ gp140 Env were blocked with 1% BSA–0.05% Tween in PBS (PBS-T). After washing, diluted samples were added to the plates for 1 h before washing and the addition of a 1:4,000 dilution of either anti-mouse IgG conjugated to horseradish peroxidase (HRP), IgG1-HRP, or IgG2a-HRP (Southern Biotech). Standards consisted of coating ELISA plate wells with anti-mouse kappa (1:3,200) and lambda (1:3,200) light chains (Serotec, United Kingdom), blocking, washing, and then adding a standard 5-fold dilution series of purified IgG or IgA (Southern Biotech, United Kingdom) starting at 1,000 ng/ml. Samples and standards were developed by using 3,3′,5,5′-tetramethylbenzidine (TMB), and the reaction was stopped after 5 min with Stop solution (Insight Biotechnologies, United Kingdom). The absorbance was read on a SpectraMax Plus ELISA plate reader (Molecular Devices) with SoftMax Pro 4.7.1 software.

The avidity indices of serum samples were determined by their antibody-antigen binding resistance to 8 M urea. Serum samples were prediluted to give an OD at 450 nm (OD_450_) readout of between 1.0 and 1.5 in an ELISA and were added to plates coated with SIV_mac239_ gp140 Env. Plates were then washed three times with either PBS-T or 8 M urea in PBS-T, before incubation with anti-mouse IgG-HRP. Samples were developed with TMB as described above. The avidity index was calculated as the percentage of the OD_450_ of urea-treated samples/OD_450_ of PBS-T-treated samples.

### SIV neutralization assay.

SIV-specific neutralization assays were performed as previously described, using the TZM.bl cell neutralization assay (30). Briefly, mouse serum was serially diluted and incubated for 1 h with the tier 1A neutralization-sensitive pseudotype virus SIVmac251.TCLA.15 and was subsequently added to TZM.bl cells. A pseudotyped virus expressing the envelope gene of murine leukemia virus (MuLV) was used as a negative control. After 48 h, cells were lysed, and relative luminescence units were quantified. The 50% infective dose (ID_50_) was calculated as the serum concentration that reduced relative luminescence units by 50% relative to the values for a no-serum control well. Pseudotyped viruses were prepared as previously described (31).

### Germinal center B cell staining.

Single-cell suspensions of iliac LN mononuclear cells were blocked with TruStain fcX (anti-mouse CD16/CD32) antibodies (BioLegend) for 10 min at 4°C. Cells were washed and stained for 30 min at 4°C with anti-CD3ε (145-2C11), anti-CD19 (6D5), anti-Fas (15A7), peanut agglutinin (PNA; Vector Laboratories), anti-IgM (RMM-1), and anti-IgD (11-26c.2a). Dead cells were excluded by the use of a vital exclusion dye (Life Technologies). All antibodies were purchased from BD Biosciences, Affymetrix, or BioLegend, unless noted otherwise. Germinal center B cells were identified by flow cytometry as Fas^+^ PNA^+^ CD19^+^, as previously described (32). Samples were acquired on an LSR II flow cytometer (BD Biosciences) and analyzed by using FlowJo v9.8.3 (TreeStar).

### Quantification of antigen-specific CD4^+^ T cells.

SIV Env-specific CD4 T cells were characterized as previously described (33). Briefly, 1 × 10^6^ to 2 × 10^6^ splenic and iliac LN mononuclear cells were stimulated for 5 h with 1 μg/ml of an overlapping SIV_mac239_ Env peptide pool (NIH AIDS Reagent Program) in the presence of GolgiStop and GolgiPlug (BD Biosciences). Cells were washed three times in autoMACS rinsing solution (Miltenyi Biotec) and stained with vital exclusion dye (Life Technologies) for 10 min at 4°C. Cells were again washed and treated with Cytofix/Cytoperm (BD Biosciences) for 20 min at 4°C. All subsequent washes and stainings were performed by using BD Perm/Wash (BD Biosciences). Cells were incubated with an interleukin-21 (IL-21) receptor (IL-21R)/Fc fusion protein (R&D Systems) for 30 min at 4°C, washed three times, and stained with goat anti-human Fcγ antibody (Jackson ImmunoResearch Laboratories) for 30 min at 4°C. Cells were subsequently washed three times and stained with anti-CD4 (RM4-5), anti-CD8a (53-6.7), anti-CD44 (IM7), anti-gamma interferon (IFN-γ) (XMG1.2), and anti-IL-2 (JES6-5H4) for 30 min at 4°C. Samples were washed three additional times, fixed in 2% formaldehyde, and stored at 4°C. Samples were acquired on an LSR II flow cytometer (BD Biosciences) and analyzed by using FlowJo v9.8.3 (TreeStar).

### Statistics.

Mann-Whitney U tests were performed with a significance cutoff of a *P* value of <0.05. Correlation analysis was performed by using the Spearman test.

## RESULTS

### Ad vector-elicited antibody responses are delayed following depletion of CD4^+^ T cells at immunization.

**FIG 1:**
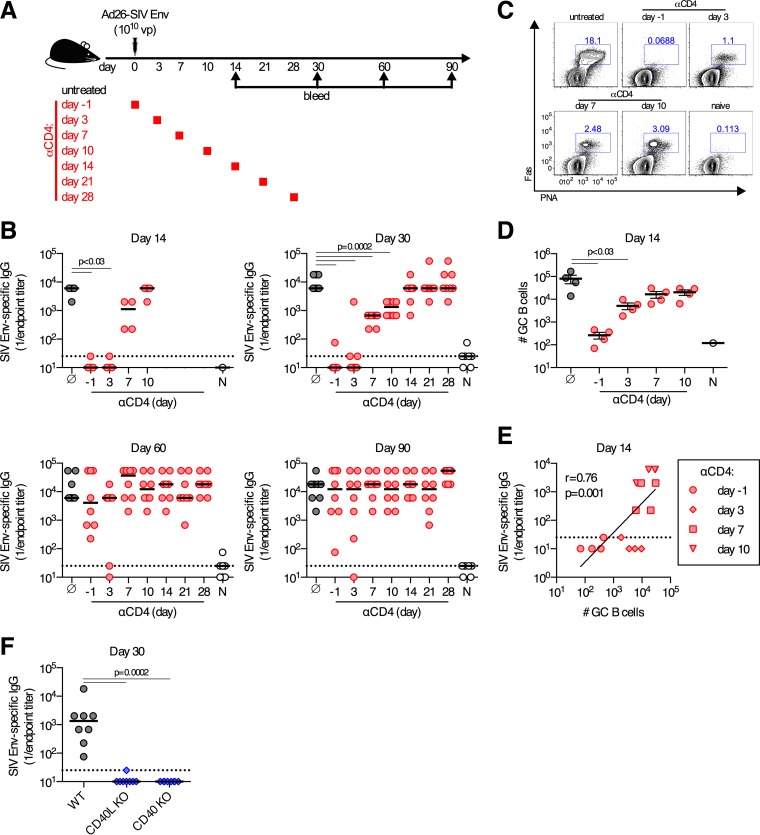
Delayed development of antibody responses following depletion of CD4^+^ T cells. (A) C57BL/6 mice were depleted of CD4^+^ T cells at the indicated days or left untreated and immunized intramuscularly with 10^10^ vp of Ad26-SIV Env. (B) SIV Env-specific serum-binding antibody titers at the indicated days postimmunization. Gray, no anti-CD4 treatment; red, anti-CD4 given on the indicated day; white, naive animals. (C and D) Representative flow cytometry plots (C) and absolute numbers (D) of germinal center (GC) B cell responses in the iliac LNs on day 14 postimmunization, with gating on CD3ε^−^ CD19^+^ cells. (E) Spearman correlation analysis of the number of germinal center B cells in iliac LNs and serum SIV Env-specific binding antibody titers on day 14 postimmunization in mice treated with anti-CD4 at the days indicated. (F) SIV Env-specific serum-binding antibody titers in wild-type (WT), CD40L KO, or CD40 KO mice immunized with 10^9^ vp of Ad26-SIV Env. Each dot represents an individual mouse, and the line is the median (B and F) or the mean ± the standard error of the mean (D). The horizontal dotted line denotes the limit of detection of the assay (for panel B, *n* = 4/group from one experiment [day 14] or *n* = 8/group pooled from two experiments [days 30, 60, and 90]; for panel D, *n* = 4/group from one experiment; for panel F, *n* = 8/group pooled from two experiments).

We first sought to determine the time at which CD4^+^ T cell help is required for the induction of transgene antigen-specific antibody responses by Ad vector vaccination. To accomplish this, C57BL/6 mice were immunized i.m. with 10^10^ vp of Ad26-SIV Env and depleted of CD4^+^ T cells by the administration of anti-CD4 antibody GK1.5 on either day −1, day 3, day 7, day 10, day 14, day 21, or day 28 postimmunization or left untreated, as a control (Fig. 1A). In untreated control mice, robust SIV Env-specific serum antibody titers were detected on day 14 postimmunization and were maintained for at least 90 days (Fig. 1B). Depletion of CD4^+^ T cells prior to immunization or on day 3 postimmunization resulted in nearly undetectable Env-specific antibody responses at days 14 and 30 postimmunization (Fig. 1B). Depletion of CD4^+^ T cells on day 7 or 10 postimmunization had progressively less of an impact on Env-specific antibody titers measured on day 30, and depletion of CD4^+^ T cells on or after day 14 postimmunization had no impact on antibody titers measured on day 30 (Fig. 1B). As expected, the decrease in antibody titers observed following the depletion of CD4^+^ T cells was highly correlated with reductions in the frequencies of germinal center B cells in the iliac (draining) lymph nodes (LNs) on day 14 postimmunization (*r* = 0.76 by Spearman test; *P* = 0.001) (Fig. 1C to E). Signaling by CD4^+^ T cells through CD40 is a well-described mechanism by which CD4^+^ T cells promote antibody responses (13). We confirmed that CD40 was an important signaling pathway for antigen-specific antibody responses following Ad vector immunization, as the absence of CD40L or CD40 resulted in a complete abolishment of Env-specific antibody responses on day 30 postimmunization (Fig. 1F). Thus, following Ad vector immunization, induction of antigen-specific antibody responses requires CD4^+^ T cell help to promote germinal center responses via CD40-derived signals, consistent with data obtained by using other experimental systems (34).

We next sought to determine whether CD4^+^ T cells regulated the maintenance of Ad vector-induced antibody responses. Unexpectedly, when Env-specific antibody responses were measured on days 60 and 90 postimmunization, we observed no significant difference in antibody titers between mice that had been previously depleted of CD4^+^ T cells and untreated control animals (Fig. 1B, bottom). Despite the lack of detectable serum antibody responses on day 30 postimmunization in mice depleted of CD4^+^ T cells prior to immunization, these same animals developed Env-specific antibody titers of a magnitude equivalent to that in untreated control mice by day 60 postimmunization. We define the antibody responses that develop on day 60 postimmunization after depletion of CD4^+^ T cells as “delayed antibody responses.” Similar delayed Env-specific antibody responses were also observed in mice depleted of CD4^+^ T cells at between days 3 and 10 postimmunization. Thus, depletion of CD4^+^ T cells immediately prior to, or after, immunization resulted in a delay but did not prevent the induction of Env-specific antibody responses.

### Delayed antibody responses following CD4^+^ T cell depletion are functional.

**FIG 2:**
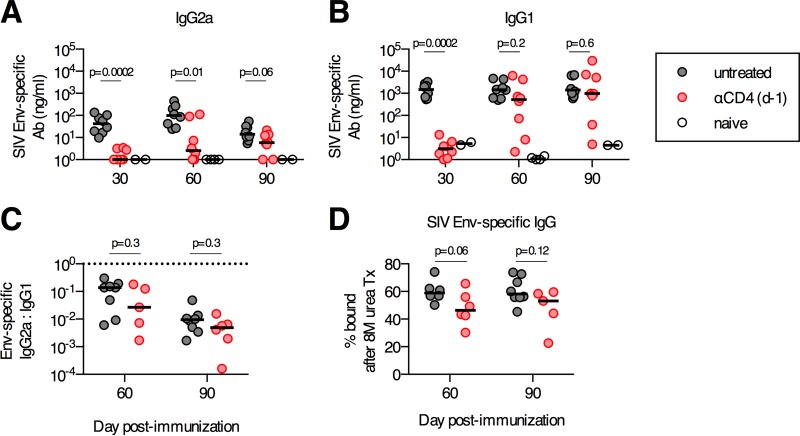
Transient depletion of CD4^+^ T cells at priming does not alter the isotype distribution or the antigen-binding avidity of delayed antibody (Ab) responses. C57BL/6 mice were depleted of CD4^+^ T cells or left untreated and immunized intramuscularly with 10^10^ vp of Ad26-SIV Env. (A and B) Concentrations of serum SIV Env-specific IgG2a (A) and IgG1 (B). (C) Ratios of SIV Env-specific IgG2a to IgG1 serum antibody titers. The horizontal dotted line denotes a ratio of 1. (D) Avidity of SIV Env-specific serum antibodies as determined by a urea disruption assay. Each dot represents an individual mouse, and the medians are indicated by the line (*n* = 5 to 8/group pooled from two experiments).

We sought to determine whether the delayed antibody responses observed after day 60 in mice depleted of CD4^+^ T cells prior to immunization are functionally normal. We first assessed if the delayed antibody responses exhibited alterations in their isotype proportions. The concentration of IgG2a isotype Env-specific antibodies increased from days 30 to 90 postimmunization in anti-CD4-treated mice, and by day 90, only a statistical trend for differences in antibody concentrations in anti-CD4-treated versus untreated mice was observed (*P* = 0.06) (Fig. 2A). Similarly, IgG1 isotype Env-specific antibody concentrations increased in anti-CD4-treated mice between days 30 and 90 and reached antibody concentrations equivalent to those in untreated control mice by day 60 (untreated median of 1,924 ng/ml and anti-CD4 median of 1,543 ng/ml; *P* = 0.2) (Fig. 2B). On both days 60 and 90 postimmunization, the ratio of IgG2a to IgG1 isotype antibodies was equivalent between the anti-CD4-treated and untreated groups (*P* = 0.3) (Fig. 2C). Finally, we sought to determine if the delayed antibody response that developed following the depletion of CD4^+^ T cells would have reduced antigen-binding avidity, as measured by using a urea disruption assay (28). Env-specific antibodies in anti-CD4-treated and untreated mice on day 60 or 90 postimmunization displayed no significant differences in Env-binding avidity as determined by urea disruption ELISAs (Fig. 2D). Thus, the delayed antibody responses exhibited no significant abnormalities in their isotypes or binding avidities.

**FIG 3:**
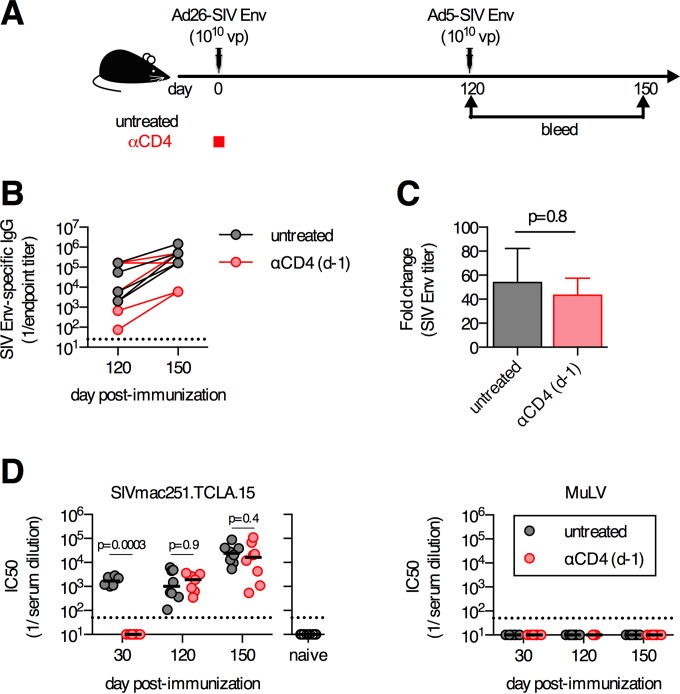
Boosting capacity and functional neutralization capacity of delayed antibody responses that develop following transient CD4^+^ T cell depletion. (A) C57BL/6 mice were depleted of CD4^+^ T cells or left untreated, immunized intramuscularly with 10^10^ vp of Ad26-SIV Env, and boosted 4 months postprime with 10^10^ vp of Ad5-SIV Env. (B) SIV Env-specific antibody titers prior to and following boosting immunization. (C) Fold change in SIV Env-specific antibody responses pre- to postboost. (D) Serum 50% neutralization capacity (IC_50_) of tier 1A SIVmac251.TCLA.15 Env-expressing pseudoviruses or a MuLV negative-control pseudovirus. Each dot represents an individual mouse, and the line indicates the mean ± the standard error of the mean (C) or the median (D). The horizontal dotted line denotes the limit of detection for the assay (*n* = 6 to 8/group pooled from two experiments).

To further investigate the functionality of these delayed antibody responses, we assessed whether these responses could expand after a boosting immunization. C57BL/6 mice were immunized i.m. with 10^10^ vp of Ad26-SIV Env and depleted of CD4^+^ T cells on day −1 or left untreated as a control (Fig. 3A). Four months after the primary immunization, mice were boosted i.m. with 10^10^ vp of Ad5-SIV Env (Fig. 3A). One month after the boosting immunization, the anti-CD4 antibody-treated and untreated mice had equivalent median SIV Env-specific endpoint titers (Fig. 3B), which reflected equivalent mean fold expansions of 54-fold and 43-fold, respectively (*P* = 0.8) (Fig. 3C). As a final measure of functional capacity, we assessed the ability of these delayed antibodies to neutralize an SIV Env-expressing pseudovirus. No SIV-specific neutralizing antibodies in anti-CD4-treated mice were detected at 1 month postimmunization (Fig. 3D), which is consistent with the lack of Env-specific binding antibodies at this time point (Fig. 1B). However, by 4 months postimmunization, anti-CD4-treated mice had median neutralizing antibody titers that were not significantly different from those in undepleted vaccinated mice (*P* = 0.9) (Fig. 3D). In both groups, neutralizing antibody titers increased following the boosting immunization, and again, no differences between the two groups were observed (Fig. 3D). Collectively, these data demonstrate that the delayed antibody responses that developed following depletion of CD4^+^ T cells at the time of primary Ad vector immunization have no detectable defects in boosting capacity, the ability to acquire functional neutralization capacity, isotype proportions, or antigen-binding avidity.

### Continuous absence of CD4^+^ T cells prevents development of antibody responses.

**FIG 4:**
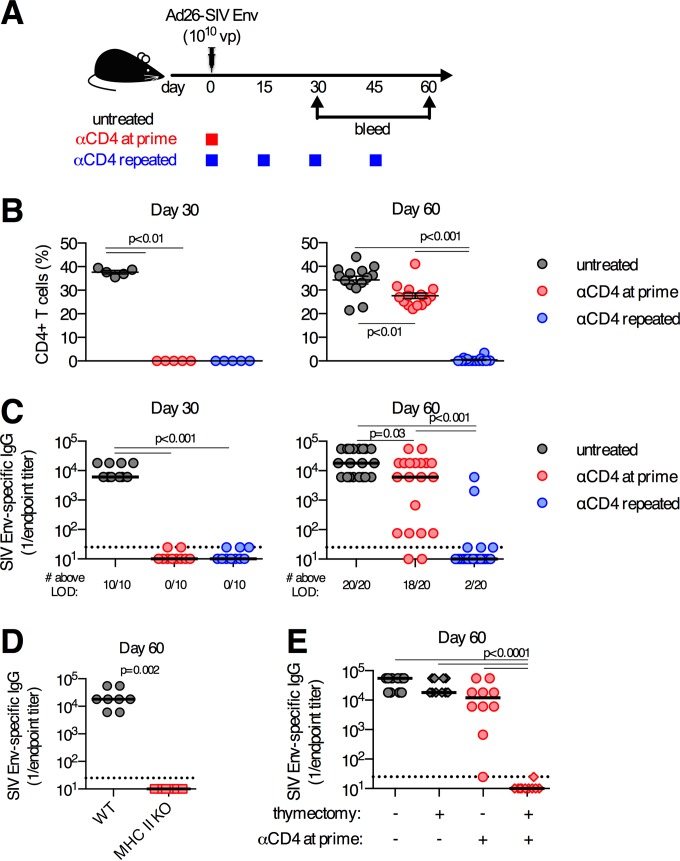
Repeated depletion of CD4^+^ T cells prevents development of delayed antibody responses. (A) C57BL/6 mice were depleted of CD4^+^ T cells a single time (anti-CD4 at prime), depleted of CD4^+^ T cells repeatedly (anti-CD4 repeated), or left untreated and immunized intramuscularly with 10^10^ vp of Ad26-SIV Env. (B) Frequency of CD4 T cells in iliac (draining) LNs. (C) SIV Env-specific serum-binding antibody titers. (D) Serum SIV Env-specific antibody titers on day 60 postimmunization from wild-type or MHC-II KO mice immunized with 10^10^ vp of Ad26-SIV Env. (E) Serum SIV Env-specific antibody titers on day 60 postimmunization from mice that underwent adult thymectomy, or not, and were treated with anti-CD4 antibody, or not, as indicated. Each dot represents an individual mouse, and the line indicates the mean ± the standard error of the mean (B) or median (C to E). The horizontal dotted line denotes the limit of detection (LOD) for the assay (for panel B, *n* = 5/group from one experiment [day 30] or *n* = 20/group pooled from four experiments [day 60]; for panel C, *n* = 10/group pooled from two experiments [day 30] or *n* = 20/group pooled from four experiments [day 60]; for panel D, *n* = 8/group pooled from two experiments; for panel E, *n* = 10/group pooled from two experiments).

We hypothesized that the development of delayed antibody responses following CD4^+^ T cell depletion reflected the transient nature of CD4^+^ T cell depletion following a single regimen of anti-CD4 antibody treatment. To test this, C57BL/6 mice were immunized i.m. with 10^10^ vp of Ad26-SIV Env and divided into three experimental groups: (i) mice treated with anti-CD4 antibody on day −1 (anti-CD4 at prime), (ii) mice treated with anti-CD4 antibody on day −1 and again every 14 to 21 days (anti-CD4 repeated), and (iii) untreated controls (Fig. 4A). Administration of anti-CD4 antibody at prime resulted in the complete depletion of CD4^+^ T cells for at least 30 days, but the CD4^+^ T cell population had largely recovered by day 60 (Fig. 4B). The recovery of the CD4^+^ T cell compartment coincided with the development of SIV Env-specific antibody responses (*r* = 0.68 by Spearman test; *P* = 0.0009) (data not shown and Fig. 4C). In contrast, repeated administration of anti-CD4 antibody maintained the depletion of CD4^+^ T cells for at least 60 days (Fig. 4B). The repeated administration of anti-CD4 antibody prevented the development of SIV Env-specific antibody responses in 18 of 20 mice by day 60 postimmunization (*P* < 0.001) (Fig. 4C). Consistent with the observation that repeated administration of anti-CD4 antibody prevented the development of a delayed antibody response, MHC-II KO mice, which permanently lack CD4^+^ T cells, had no SIV Env-specific antibody responses on day 60 postimmunization (Fig. 4D). Thus, the recovery of CD4^+^ T cells following anti-CD4 antibody treatment is necessary for the development of these delayed antibody responses.

We next tested the possibility that the development of delayed antibody responses is due to incomplete depletion of CD4^+^ T cells following treatment, thus resulting in signals from residual primed antigen-specific CD4^+^ T cells. To test this, adult C57BL/6 mice were thymectomized and depleted of CD4^+^ T cells prior to immunization with Ad26-SIV Env. A single administration of the anti-CD4 antibody to thymectomized mice results in a permanent depletion of CD4^+^ T cells, due to an inability to generate new CD4^+^ T cells (data not shown). On day 60 postimmunization, no SIV Env-specific antibodies were detected in thymectomized mice treated at priming with anti-CD4 antibody (Fig. 4E). Thus, our data show that any residual CD4^+^ T cells are insufficient for the generation of a delayed antibody response. Moreover, our data further highlight that *de novo* generation of CD4^+^ T cells by the thymus is required for the delayed antibody response to develop. Collectively, these data demonstrate that the delayed antibody response that develops following anti-CD4 antibody treatment requires thymus-driven reconstitution of the CD4^+^ T cell compartment.

### CD4^+^ T cell rebound following transient depletion induces normal germinal center responses.

**FIG 5:**
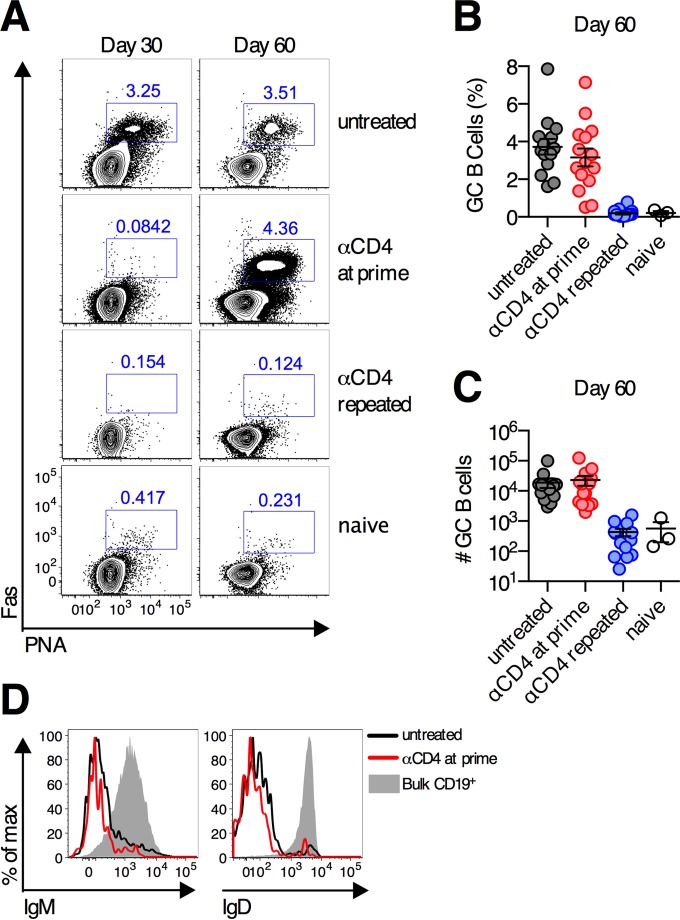
Germinal center B cell responses develop following transient depletion of CD4^+^ T cells. C57BL/6 mice were depleted of CD4 T cells a single time (anti-CD4 at prime) (red circles), depleted of CD4 T cells repeatedly (anti-CD4 repeated) (blue circles), or left untreated (gray circles) and immunized intramuscularly with 10^10^ vp of Ad26-SIV Env. (A to C) Representative flow cytometry plots (A), group average percentages (B), and absolute numbers (C) of germinal center B cells in the iliac (draining) LNs on day 60 postimmunization. (D) Expression of IgM and IgD on germinal center B cells on day 60 postimmunization. Each dot represents an individual mouse, and the means ± standard errors of the means are indicated by a line (*n* = 15/group pooled from three experiments).

As CD4^+^ T cells were critical for promoting the formation of germinal center responses following Ad vector immunization (Fig. 1), we hypothesized that when CD4^+^ T cells recovered following transient depletion, *de novo* germinal center responses would be induced. On day 30 postimmunization, no germinal center B cells were detected in the iliac lymph nodes of Ad26-SIV Env-immunized mice treated with anti-CD4 antibody at priming (Fig. 5A). However, by day 60 postimmunization, when the frequency of CD4^+^ T cells had returned to near-basal levels in these animals, robust germinal center responses were observed. These responses were equivalent in frequency and absolute number of cells to the germinal center responses measured in untreated control mice (Fig. 5A to C). These germinal center B cells also displayed normal downregulation of IgM and IgD (Fig. 5D). Recovery of CD4^+^ T cells was required for the induction of these delayed germinal center responses, as sustained depletion of CD4^+^ T cells prevented germinal centers from developing (Fig. 5A to C). Thus, recovery of CD4^+^ T cells results in the generation and expansion of germinal centers and the development of the delayed antibody response.

### Expansion of antigen-specific CD4^+^ T cells occurs following transient CD4^+^ T cell depletion.

**FIG 6:**
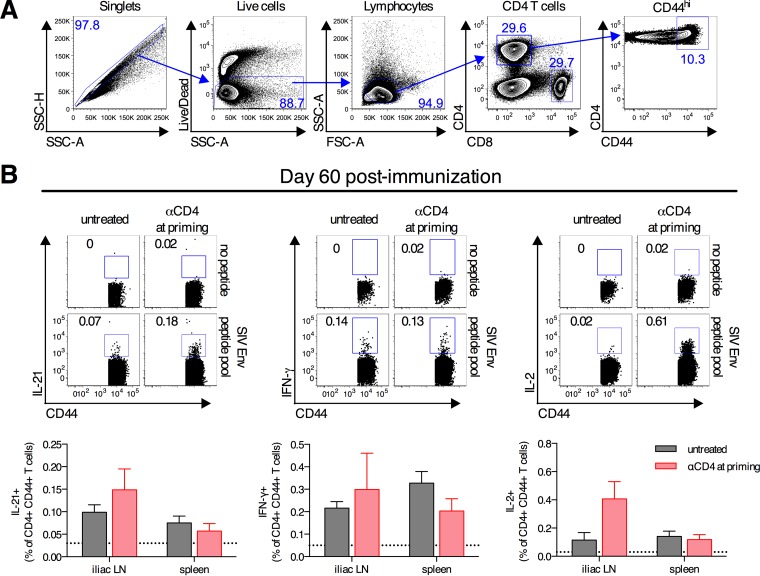
Development of antigen-specific CD4^+^ T cell responses after transient depletion of CD4^+^ T cells. (A) Gating scheme for characterization of antigen-specific CD4^+^ T cells by intracellular cytokine staining. SSC, side scatter; FSC, forward scatter. (B) C57BL/6 mice were depleted of CD4^+^ T cells at immunization or left untreated and immunized intramuscularly with 10^10^ vp of Ad26-SIV Env. Spleens and iliac (draining) LNs were collected on day 60 postimmunization, and intracellular cytokine staining was performed to detect the production of IL-21, IFN-γ, and IL-2 by CD44^+^ CD4^+^ T cells following stimulation with an overlapping SIV Env peptide pool. Means ± standard errors of the means are shown. The horizontal dotted line denotes the limit of detection for the assay (*n* = 10/group pooled from two experiments).

We next sought to characterize the recovering CD4^+^ T cells to determine if any of the cells were specific for the SIV Env antigen. To test this, on day 60 postimmunization, CD4^+^ T cells from the spleen and iliac LNs were stimulated with an overlapping SIV Env peptide pool, and cytokine production was measured by intracellular cytokine staining (Fig. 6A). CD4^+^ T cells from both untreated control mice and mice treated with anti-CD4 antibody at priming produced modest amounts of IL-21, IFN-γ, and IL-2 in response to SIV Env peptide stimulation (Fig. 6B). Antigen-specific CD4^+^ T cells were detected in both the spleen and iliac LNs of mice from both groups. IL-21-producing CD4^+^ T cells were identified, and as CD4^+^ T cell-derived IL-21 is a critical cytokine for germinal center responses (35), it likely partially explains how delayed germinal center responses are induced. These data demonstrate that in the context of Ad vector immunization following transient CD4^+^ T cell depletion, antigen-specific CD4^+^ T cells expand upon recovery.

### Development of delayed antibody responses occurs following soluble protein immunization.

**FIG 7:**
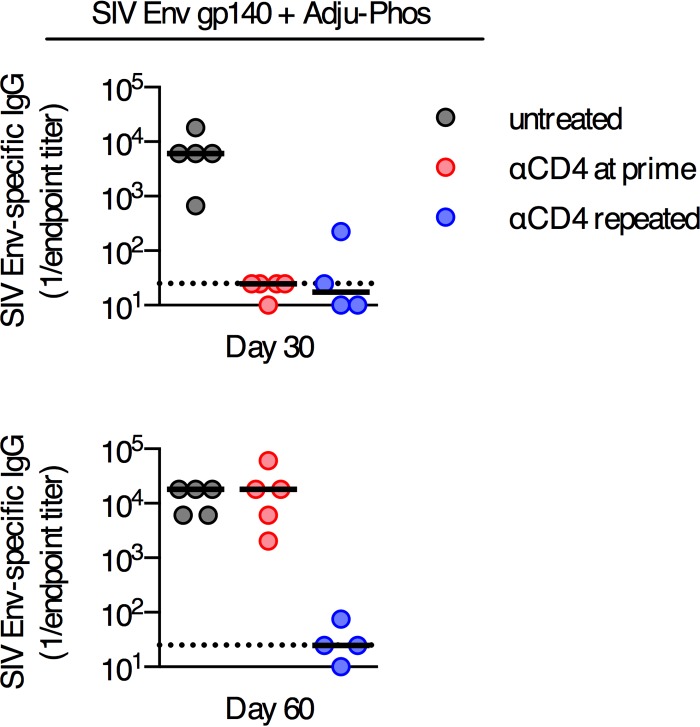
Delayed antibody responses develop following immunization with an adjuvanted soluble protein formulated in Adju-Phos but not following immunization with a replication-incompetent poxvirus vector. SIV Env-specific serum antibody titers in C57BL/6 mice immunized intramuscularly with 50 μg of SIV Env gp140 plus Adju-Phos and depleted of CD4^+^ T cells at the time of immunization (anti-CD4 at prime) or continuously depleted of CD4^+^ T cells (anti-CD4 repeated) or mice left untreated were determined. Each dot represents an individual mouse, and the line is the median. The horizontal dotted line denotes the limit of detection for the assay (*n* = 4 to 5/group from one experiment).

We sought to identify other immunization regimens where delayed antibody responses may occur following transient depletion of CD4^+^ T cells. Therefore, we tested adjuvanted soluble protein immunogens as an alternate vaccine platform. C57BL/6 mice were immunized i.m. with 50 μg of trimeric SIV Env gp140 protein formulated in 100 μg of Adju-Phos adjuvant, and mice were either left untreated, depleted of CD4^+^ T cells at priming, or repeatedly depleted of CD4^+^ T cells (Fig. 7). This immunization regimen induced robust Env-specific titers at day 30 postimmunization in untreated control mice (Fig. 7), and titers were maintained to day 60. In mice transiently depleted of CD4^+^ T cells, no Env-specific antibody responses were detected on day 30 postimmunization, but by day 60 postimmunization, these animals had titers equivalent to those in untreated control animals (Fig. 7). Continuous depletion of CD4^+^ T cells by repeated administration of anti-CD4 antibody prevented the development of Env-specific antibodies on day 60 (Fig. 7). These data demonstrate that a delayed antibody response can occur in the context of multiple immunization regimens and is not a unique characteristic of immunization with replication-incompetent Ad vectors.

## DISCUSSION

In this study, we demonstrate that the induction of transgene antigen-specific antibody responses by vaccination can be temporally separated from the time of immunization by the transient depletion of CD4^+^ T cells. CD4^+^ T cell help is required for the induction of antibody responses. However, the transient absence of CD4^+^ T cells at the time of Ad vector immunization delays the development of an antibody response only until the time at which the CD4^+^ T cell population returns, and we identify no functional defects in these delayed antibody responses once they develop. The development of a delayed antibody response coincides with the expansion of antigen-specific CD4^+^ T cells. This is unexpected because we previously demonstrated that the absence of CD4^+^ T cells at the time of Ad vector immunization immediately and irreversibly impairs the development of functional CD8^+^ T cell responses (23; Provine et al., submitted). Thus, while CD4^+^ T cell help is critical for the induction of both antibody and CD8^+^ T cell responses following Ad vector vaccination, the timing and mechanisms of CD4^+^ T cell help are profoundly different. These findings have implications for understating the biology of antibody responses elicited by vaccination.

CD4^+^ T cells have many well-established roles in regulating antibody responses against an array of antigens (3). One key aspect of this process is the sustained provision of positive signals to responding B cells by CD4^+^ T cells (8, 13, 14). In those previous studies, which all utilized soluble protein antigens, the need for CD4^+^ T cell help was absolute at the time of antigen exposure and waned gradually. Subsequent work directly visualized CD4^+^ T cell-B cell contacts for >1 week postimmunization (12), which is in line with the timing determined by CD4^+^ T cell depletion studies. Consistent with these findings, the induction of robust antibody responses by Ad vector immunization requires sustained CD4^+^ T cell help for between 10 and 14 days following immunization (Fig. 1). Thus, prolonged, gradually waning CD4^+^ T cell help to B cells appears to be a highly generalizable trait.

However, depletion of CD4^+^ T cells at the time of antigen administration was initially suggested as a means to induce B cell tolerance, since antigen-specific antibody responses do not develop even after the CD4^+^ T cell population recovers (4–9). In contrast to those reports, we demonstrate that transient CD4^+^ T cell depletion at the time of intramuscular immunization with an Ad vector or a protein formulated with alum did not induce tolerance to the vaccine antigen. Instead, once the CD4^+^ T cell compartment is reconstituted, robust antigen-specific antibody responses develop without the need to experimentally readminister the antigen, and these responses appear functionally indistinguishable by our measures from the response induced if CD4^+^ T cells are present at the time of immunization. Thus, while CD4^+^ T cell help is required for the induction of antigen-specific antibody responses elicited by Ad vectors and soluble proteins formulated in an alum-based adjuvant, this help need not be provided at the time of immunization.

Several studies have demonstrated that, in principle, CD4^+^ T cell help can be provided after initial antigen exposure (7, 36–38), but in those previous reports, additional experimental stimulation of CD4^+^ T cells was required to induce antibody responses. Aubert and colleagues (7) demonstrated that adoptive transfer of naive lymphocytic choriomeningitis virus (LCMV)-specific CD4^+^ T cells into mice transiently depleted of CD4^+^ T cells at the time of LCMV clone 13 infection results in the generation of LCMV-specific antibody responses. The other three studies (36–38) all utilized self-antigen-specific B cell receptor (BCR) transgenic mouse models. In such a system, cognate B cell antigens are continuously present, and therefore, any provision of CD4^+^ T cell help can be seen as being after the time of initial antigen exposure. In all three reports, experimental provision of CD4^+^ T cell help induced the development of autoantibody responses. However, in all of those previous studies, antigen-specific CD4^+^ T cells were adoptively transferred into recipient animals and were experimentally activated prior to or after transfer. From those studies, it remains unclear if CD4^+^ T cell help can occur at a time separated from the time of initial antigen exposure without experimental manipulation and reactivation of the CD4^+^ T cell compartment. In this study, we identify antigen-specific cells within the recovering CD4^+^ T cell population without the need for experimental manipulation, and the presence of these cells likely predicates the development of the delayed antibody response. Thus, we demonstrate for the first time, to our knowledge, that endogenous CD4^+^ T cell responses can provide help to B cells at a time separated from the time of initial antigen exposure.

Optimal priming of a T cell response requires T cell receptor (TCR) engagement, costimulation, and proinflammatory cytokines (1). The induction of a delayed antibody response requires *de novo* thymic production of naive CD4^+^ T cells (Fig. 4), and it takes >30 days for these cells to recover following depletion. Thus, for priming of CD4^+^ T cells to occur, Ad vector immunization must induce sufficient expression of the necessary priming pathways for at least 30 days postimmunization. It was previously reported that Ad vectors induce the necessary signals to induce naive CD8^+^ T cell proliferation for at least 30 days postimmunization (39, 40). We extend these findings by demonstrating not only that Ad vectors provide the necessary signals to induce T cell proliferation for more than a month postimmunization but also that Ad vectors induce all of the signals necessary to induce a functional CD4^+^ T helper response for at least a month postimmunization. Thus, despite lacking the capacity to replicate, Ad vectors and appropriate soluble protein-based immunization regimens can induce functional T cell responses at a time markedly separated from the time of immunization.

How B cell priming occurs with regard to a delayed antibody response remains unclear. Even in response to T cell-dependent antigens, the initial activation of naive B cells by cognate binding of antigen to the BCR occurs prior to the interaction with T cells (41–43). However, a failure to immediately receive T cell help results in the anergy or apoptosis of these activated B cells (44–47). Thus, it is possible that the delayed antibody response that occurs following transient depletion of CD4^+^ T cells is the result of these antigen-experienced B cells receiving the necessary activation signals following reconstitution of the CD4^+^ T cell compartment. Alternatively, the delayed antibody response may instead involve B cells that recognize antigen and become activated at a time more contemporaneous with the recovery of CD4^+^ T cells. In this study, the vaccine doses used (10^10^ vp of the Ad vector or 50 μg of protein) are on the high end of doses used in mice but are not outside accepted dose ranges for these immunogens in mice (13, 48–51), and these high antigen levels might allow sufficient antigen persistence to prime B cells at a time coinciding with the recovery of CD4^+^ T cells. Future experiments will be required to distinguish between these two possibilities and will enhance our understanding of this phenomenon.

In conclusion, we have shown the role of CD4^+^ T cell responses in helping to elicit serum antigen-specific antibody responses following immunization with nonreplicating Ad vectors and adjuvanted soluble protein immunogens. Our data demonstrate that antibody responses can be induced at a time separated from the time of immunization by manipulation of CD4^+^ T cells, and this may be a useful approach to specifically control the timing of development of antibody responses. These findings demonstrate that the provision of critical CD4^+^ T cell-derived help signals to B cells can occur substantially after the time of initial antigen exposure. Thus, the temporal regulation of Ad vector-elicited antibody responses by CD4^+^ T cells is completely distinct from the temporal regulation of Ad vector-elicited CD8^+^ T cell responses (23; Provine et al., submitted). These data identify a previously unappreciated dynamic nature of the timing of CD4^+^ T cell help for the induction of antibody responses.

## References

[1] Swain SL, McKinstry KK, Strutt TM 2012 Expanding roles for CD4+ T cells in immunity to viruses. Nat Rev Immunol 12:136–148. doi:10.1038/nri3152. 22266691 PMC3764486

[2] Fulcher DA, Lyons AB, Korn SL, Cook MC, Koleda C, Parish C, Fazekas de St Groth B, Basten A 1996 The fate of self-reactive B cells depends primarily on the degree of antigen receptor engagement and availability of T cell help. J Exp Med 183:2313–2328. doi:10.1084/jem.183.5.2313. 8642340 PMC2192557

[3] Victora GD, Nussenzweig MC 2012 Germinal centers. Annu Rev Immunol 30:429–457. doi:10.1146/annurev-immunol-020711-075032. 22224772

[4] Benjamin RJ, Waldmann H 1986 Induction of tolerance by monoclonal antibody therapy. Nature 320:449–451. doi:10.1038/320449a0. 2938014

[5] Goronzy J, Weyand CM, Fathman CG 1986 Long-term humoral unresponsiveness in vivo, induced by treatment with monoclonal antibody against L3T4. J Exp Med 164:911–925. doi:10.1084/jem.164.3.911. 3091757 PMC2188395

[6] Goronzy JJ, Weyand CM 1989 Persistent suppression of virus-specific cytotoxic T cell responses after transient depletion of CD4+ T cells in vivo. J Immunol 142:4435–4440. 2524530

[7] Aubert RD, Kamphorst AO, Sarkar S, Vezys V, Ha SJ, Barber DL, Ye L, Sharpe AH, Freeman GJ, Ahmed R 2011 Antigen-specific CD4 T-cell help rescues exhausted CD8 T cells during chronic viral infection. Proc Natl Acad Sci U S A 108:21182–21187. doi:10.1073/pnas.1118450109. 22160724 PMC3248546

[8] Vieira P, Rajewsky K 1990 Persistence of memory B cells in mice deprived of T cell help. Int Immunol 2:487–494. doi:10.1093/intimm/2.6.487. 2150759

[9] Biasi G, Facchinetti A, Panozzo M, Zanovello P, Chieco-Bianchi L, Collavo D 1991 Moloney murine leukemia virus tolerance in anti-CD4 monoclonal antibody-treated adult mice. J Immunol 147:2284–2289. 1833453

[10] Lane P, Traunecker A, Hubele S, Inui S, Lanzavecchia A, Gray D 1992 Activated human T cells express a ligand for the human B cell-associated antigen CD40 which participates in T cell-dependent activation of B lymphocytes. Eur J Immunol 22:2573–2578. doi:10.1002/eji.1830221016. 1382991

[11] McIntosh JH, Cochrane M, Cobbold S, Waldmann H, Nathwani SA, Davidoff AM, Nathwani AC 2012 Successful attenuation of humoral immunity to viral capsid and transgenic protein following AAV-mediated gene transfer with a non-depleting CD4 antibody and cyclosporine. Gene Ther 19:78–85. doi:10.1038/gt.2011.64. 21716299 PMC3526978

[12] Shulman Z, Gitlin AD, Targ S, Jankovic M, Pasqual G, Nussenzweig MC, Victora GD 2013 T follicular helper cell dynamics in germinal centers. Science 341:673–677. doi:10.1126/science.1241680. 23887872 PMC3941467

[13] Han S, Hathcock K, Zheng B, Kepler TB, Hodes R, Kelsoe G 1995 Cellular interaction in germinal centers. Roles of CD40 ligand and B7-2 in established germinal centers. J Immunol 155:556–567. 7541819

[14] Finkelman FD, Holmes J, Urban JF, Paul WE, Katona IM 1989 T help requirements for the generation of an in vivo IgE response: a late acting form of T cell help other than IL-4 is required for IgE but not for IgG1 production. J Immunol 142:403–408. 2521345

[15] Finkelman FD, Holmes JM, Dukhanina OI, Morris SC 1995 Cross-linking of membrane immunoglobulin D, in the absence of T cell help, kills mature B cells in vivo. J Exp Med 181:515–525. doi:10.1084/jem.181.2.515. 7836908 PMC2191863

[16] Sullivan NJ, Hensley L, Asiedu C, Geisbert TW, Stanley D, Johnson J, Honko A, Olinger G, Bailey M, Geisbert JB, Reimann KA, Bao S, Rao S, Roederer M, Jahrling PB, Koup RA, Nabel GJ 2011 CD8(+) cellular immunity mediates rAd5 vaccine protection against Ebola virus infection of nonhuman primates. Nat Med 17:1128–1131. doi:10.1038/nm.2447. 21857654

[17] Barnes E, Folgori A, Capone S, Swadling L, Aston S, Kurioka A, Meyer J, Huddart R, Smith K, Townsend R, Brown A, Antrobus R, Ammendola V, Naddeo M, O'Hara G, Willberg C, Harrison A, Grazioli F, Esposito ML, Siani L, Traboni C, Oo Y, Adams D, Hill A, Colloca S, Nicosia A, Cortese R, Klenerman P 2012 Novel adenovirus-based vaccines induce broad and sustained T cell responses to HCV in man. Sci Transl Med 4:115ra1. doi:10.1126/scitranslmed.3003155. PMC3627207 22218690

[18] Smaill F, Jeyanathan M, Smieja M, Medina MF, Thanthrige-Don N, Zganiacz A, Yin C, Heriazon A, Damjanovic D, Puri L, Hamid J, Xie F, Foley R, Bramson J, Gauldie J, Xing Z 2013 A human type 5 adenovirus-based tuberculosis vaccine induces robust T cell responses in humans despite preexisting anti-adenovirus immunity. Sci Transl Med 5:205ra134. doi:10.1126/scitranslmed.3006843. 24089406

[19] Liu J, O'Brien KL, Lynch DM, Simmons NL, La Porte A, Riggs AM, Abbink P, Coffey RT, Grandpre LE, Seaman MS, Landucci G, Forthal DN, Montefiori DC, Carville A, Mansfield KG, Havenga MJ, Pau MG, Goudsmit J, Barouch DH 2009 Immune control of an SIV challenge by a T-cell-based vaccine in rhesus monkeys. Nature 457:87–91. doi:10.1038/nature07469. 18997770 PMC2614452

[20] Barouch DH, Liu J, Li H, Maxfield LF, Abbink P, Lynch DM, Iampietro MJ, SanMiguel A, Seaman MS, Ferrari G, Forthal DN, Ourmanov I, Hirsch VM, Carville A, Mansfield KG, Stablein D, Pau MG, Schuitemaker H, Sadoff JC, Billings EA, Rao M, Robb ML, Kim JH, Marovich MA, Goudsmit J, Michael NL 2012 Vaccine protection against acquisition of neutralization-resistant SIV challenges in rhesus monkeys. Nature 482:89–93. doi:10.1038/nature10766. 22217938 PMC3271177

[21] Barouch DH, Stephenson KE, Borducchi EN, Smith K, Stanley K, McNally AG, Liu J, Abbink P, Maxfield LF, Seaman MS, Dugast A-S, Alter G, Ferguson M, Li W, Earl PL, Moss B, Giorgi EE, Szinger JJ, Eller LA, Billings EA, Rao M, Tovanabutra S, Sanders-Buell E, Weijtens M, Pau MG, Schuitemaker H, Robb ML, Kim JH, Korber BT, Michael NL 2013 Protective efficacy of a global HIV-1 mosaic vaccine against heterologous SHIV challenges in rhesus monkeys. Cell 155:531–539. doi:10.1016/j.cell.2013.09.061. 24243013 PMC3846288

[22] Barouch DH, Alter G, Broge T, Linde C, Ackerman ME, Brown EP, Borducchi EN, Smith KM, Nkolola JP, Liu J, Shields J, Parenteau L, Whitney JB, Abbink P, Ng'ang'a DM, Seaman MS, Lavine CL, Perry JR, Li W, Colantonio AD, Lewis MG, Chen B, Wenschuh H, Reimer U, Piatak M, Lifson JD, Handley SA, Virgin HW, Koutsoukos M, Lorin C, Voss G, Weijtens M, Pau MG, Schuitemaker H 2015 Protective efficacy of adenovirus/protein vaccines against SIV challenges in rhesus monkeys. Science 349:320–324. doi:10.1126/science.aab3886. 26138104 PMC4653134

[23] Provine NM, Larocca RA, Penaloza-MacMaster P, Borducchi EN, McNally A, Parenteau LR, Kaufman DR, Barouch DH 2014 Longitudinal requirement for CD4+ T cell help for adenovirus vector-elicited CD8+ T cell responses. J Immunol 192:5214–5225. doi:10.4049/jimmunol.1302806. 24778441 PMC4025612

[24] Abbink P, Lemckert AAC, Ewald BA, Lynch DM, Denholtz M, Smits S, Holterman L, Damen I, Vogels R, Thorner AR, O'Brien KL, Carville A, Mansfield KG, Goudsmit J, Havenga MJE, Barouch DH 2007 Comparative seroprevalence and immunogenicity of six rare serotype recombinant adenovirus vaccine vectors from subgroups B and D. J Virol 81:4654–4663. doi:10.1128/JVI.02696-06. 17329340 PMC1900173

[25] Chen B, Zhou G, Kim M, Chishti Y, Hussey RE, Ely B, Skehel JJ, Reinherz EL, Harrison SC, Wiley DC 2000 Expression, purification, and characterization of gp160e, the soluble, trimeric ectodomain of the simian immunodeficiency virus envelope glycoprotein, gp160. J Biol Chem 275:34946–34953. doi:10.1074/jbc.M004905200. 10944528

[26] Wherry EJ, Teichgräber V, Becker TC, Masopust D, Kaech SM, Antia R, von Andrian UH, Ahmed R 2003 Lineage relationship and protective immunity of memory CD8 T cell subsets. Nat Immunol 4:225–234. doi:10.1038/ni889. 12563257

[27] Barouch DH, Pau MG, Custers JHHV, Koudstaal W, Kostense S, Havenga MJE, Truitt DM, Sumida SM, Kishko MG, Arthur JC, Korioth-Schmitz B, Newberg MH, Gorgone DA, Lifton MA, Panicali DL, Nabel GJ, Letvin NL, Goudsmit J 2004 Immunogenicity of recombinant adenovirus serotype 35 vaccine in the presence of pre-existing anti-Ad5 immunity. J Immunol 172:6290–6297. doi:10.4049/jimmunol.172.10.6290. 15128818

[28] Mann JFS, McKay PF, Fiserova A, Klein K, Cope A, Rogers P, Swales J, Seaman MS, Combadière B, Shattock RJ 2014 Enhanced immunogenicity of an HIV-1 DNA vaccine delivered with electroporation via combined intramuscular and intradermal routes. J Virol 88:6959–6969. doi:10.1128/JVI.00183-14. 24719412 PMC4054344

[29] Badamchi-Zadeh A, McKay PF, Holland MJ, Paes W, Brzozowski A, Lacey C, Follmann F, Tregoning JS, Shattock RJ 2015 Intramuscular immunisation with chlamydial proteins induces Chlamydia trachomatis specific ocular antibodies. PLoS One 10:e0141209. doi:10.1371/journal.pone.0141209. 26501198 PMC4621052

[30] Sarzotti-Kelsoe M, Bailer RT, Turk E, Lin C-L, Bilska M, Greene KM, Gao H, Todd CA, Ozaki DA, Seaman MS, Mascola JR, Montefiori DC 2014 Optimization and validation of the TZM-bl assay for standardized assessments of neutralizing antibodies against HIV-1. J Immunol Methods 409:131–146. doi:10.1016/j.jim.2013.11.022. 24291345 PMC4040342

[31] Seaman MS, Janes H, Hawkins N, Grandpre LE, Devoy C, Giri A, Coffey RT, Harris L, Wood B, Daniels MG, Bhattacharya T, Lapedes A, Polonis VR, McCutchan FE, Gilbert PB, Self SG, Korber BT, Montefiori DC, Mascola JR 2010 Tiered categorization of a diverse panel of HIV-1 Env pseudoviruses for assessment of neutralizing antibodies. J Virol 84:1439–1452. doi:10.1128/JVI.02108-09. 19939925 PMC2812321

[32] Johnston RJ, Poholek AC, DiToro D, Yusuf I, Eto D, Barnett B, Dent AL, Craft J, Crotty S 2009 Bcl6 and Blimp-1 are reciprocal and antagonistic regulators of T follicular helper cell differentiation. Science 325:1006–1010. doi:10.1126/science.1175870. 19608860 PMC2766560

[33] Suto A, Kashiwakuma D, Kagami SI, Hirose K, Watanabe N, Yokote K, Saito Y, Nakayama T, Grusby MJ, Iwamoto I, Nakajima H 2008 Development and characterization of IL-21-producing CD4+ T cells. J Exp Med 205:1369–1379. doi:10.1084/jem.20072057. 18474630 PMC2413034

[34] Crotty S 2011 Follicular helper CD4 T cells (TFH). Annu Rev Immunol 29:621–663. doi:10.1146/annurev-immunol-031210-101400. 21314428

[35] Parrish-Novak J, Dillon SR, Nelson A, Hammond A, Sprecher C, Gross JA, Johnston J, Madden K, Xu W, West J, Schrader S, Burkhead S, Heipel M, Brandt C, Kuijper JL, Kramer J, Conklin D, Presnell SR, Berry J, Shiota F, Bort S, Hambly K, Mudri S, Clegg C, Moore M, Grant FJ, Lofton-Day C, Gilbert T, Rayond F, Ching A, Yao L, Smith D, Webster P, Whitmore T, Maurer M, Kaushansky K, Holly RD, Foster D 2000 Interleukin 21 and its receptor are involved in NK cell expansion and regulation of lymphocyte function. Nature 408:57–63. doi:10.1038/35040504. 11081504

[36] Bachmann MF, Rohrer UH, Steinhoff U, Bürki K, Skuntz S, Arnheiter H, Hengartner H, Zinkernagel RM 1994 T helper cell unresponsiveness: rapid induction in antigen-transgenic and reversion in non-transgenic mice. Eur J Immunol 24:2966–2973. doi:10.1002/eji.1830241207. 7805723

[37] Steinhoff U, Burkhart C, Arnheiter H, Hengartner H, Zinkernagel R 1994 Virus or a hapten-carrier complex can activate autoreactive B cells by providing linked T help. Eur J Immunol 24:773–776. doi:10.1002/eji.1830240343. 7907298

[38] Keech CL, Farris AD, Beroukas D, Gordon TP, McCluskey J 2001 Cognate T cell help is sufficient to trigger anti-nuclear autoantibodies in naive mice. J Immunol 166:5826–5834. doi:10.4049/jimmunol.166.9.5826. 11313427

[39] Kaufman DR, Bivas-Benita M, Simmons NL, Miller D, Barouch DH 2010 Route of adenovirus-based HIV-1 vaccine delivery impacts the phenotype and trafficking of vaccine-elicited CD8^+^ T lymphocytes. J Virol 84:5986–5996. doi:10.1128/JVI.02563-09. 20357087 PMC2876628

[40] Yang T-CT, Millar JJ, Groves TT, Grinshtein NN, Parsons RR, Takenaka SS, Wan YY, Bramson JLJ 2006 The CD8+ T cell population elicited by recombinant adenovirus displays a novel partially exhausted phenotype associated with prolonged antigen presentation that nonetheless provides long-term immunity. J Immunol 176:200–210. doi:10.4049/jimmunol.176.1.200. 16365411

[41] Liu YJ, Zhang J, Lane PJ, Chan EY, MacLennan IC 1991 Sites of specific B cell activation in primary and secondary responses to T cell-dependent and T cell-independent antigens. Eur J Immunol 21:2951–2962. doi:10.1002/eji.1830211209. 1748148

[42] Garside P, Ingulli E, Merica RR, Johnson JG, Noelle RJ, Jenkins MK 1998 Visualization of specific B and T lymphocyte interactions in the lymph node. Science 281:96–99. doi:10.1126/science.281.5373.96. 9651253

[43] Reif K, Ekland EH, Ohl L, Nakano H, Lipp M, Förster R, Cyster JG 2002 Balanced responsiveness to chemoattractants from adjacent zones determines B-cell position. Nature 416:94–99. doi:10.1038/416094a. 11882900

[44] Goodnow CC, Crosbie J, Adelstein S, Lavoie TB, Smith-Gill SJ, Brink RA, Pritchard-Briscoe H, Wotherspoon JS, Loblay RH, Raphael K 1988 Altered immunoglobulin expression and functional silencing of self-reactive B lymphocytes in transgenic mice. Nature 334:676–682. doi:10.1038/334676a0. 3261841

[45] Goodnow CC, Crosbie J, Jorgensen H, Brink RA, Basten A 1989 Induction of self-tolerance in mature peripheral B lymphocytes. Nature 342:385–391. doi:10.1038/342385a0. 2586609

[46] Hartley SB, Cooke MP, Fulcher DA, Harris AW, Cory S, Basten A, Goodnow CC 1993 Elimination of self-reactive B lymphocytes proceeds in two stages: arrested development and cell death. Cell 72:325–335. doi:10.1016/0092-8674(93)90111-3. 8431943

[47] Nemazee DA, Bürki K 1989 Clonal deletion of B lymphocytes in a transgenic mouse bearing anti-MHC class I antibody genes. Nature 337:562–566. doi:10.1038/337562a0. 2783762

[48] Darrah PA, Hegde ST, Patel DT, Lindsay RWB, Chen L, Roederer M, Seder RA 2010 IL-10 production differentially influences the magnitude, quality, and protective capacity of Th1 responses depending on the vaccine platform. J Exp Med 207:1421–1433. doi:10.1084/jem.20092532. 20530206 PMC2901071

[49] Penaloza-MacMaster P, Provine NM, Ra J, Borducchi EN, McNally A, Simmons NL, Iampietro MJ, Barouch DH 2013 Alternative serotype adenovirus vaccine vectors elicit memory T cells with enhanced anamnestic capacity compared to Ad5 vectors. J Virol 87:1373–1384. doi:10.1128/JVI.02058-12. 23152535 PMC3554181

[50] Wang Y, Bhattacharya D 2014 Adjuvant-specific regulation of long-term antibody responses by ZBTB20. J Exp Med 211:841–856. doi:10.1084/jem.20131821. 24711582 PMC4010912

[51] Hu JK, Crampton JC, Cupo A, Ketas T, van Gils MJ, Sliepen K, de Taeye SW, Sok D, Ozorowski G, Deresa I, Stanfield R, Ward AB, Burton DR, Klasse PJ, Sanders RW, Moore JP, Crotty S 2015 Murine antibody responses to cleaved soluble HIV-1 envelope trimers are highly restricted in specificity. J Virol 89:10383–10398. doi:10.1128/JVI.01653-15. 26246566 PMC4580201

